# Optical Imaging of Nonuniform Ferroelectricity and Strain at the Diffraction Limit

**DOI:** 10.1038/srep15800

**Published:** 2015-11-02

**Authors:** Ondrej Vlasin, Blai Casals, Nico Dix, Diego Gutiérrez, Florencio Sánchez, Gervasi Herranz

**Affiliations:** 1 Institut de Ciència de Materials de Barcelona (ICMAB-CSIC), Campus de la UAB, 08193 Bellaterra, Catalonia, Spain

## Abstract

We have imaged optically the spatial distributions of ferroelectricity and piezoelectricity at the diffraction limit. Contributions to the birefringence from electro-optics –linked to ferroelectricity– as well as strain –arising from converse piezoelectric effects– have been recorded simultaneously in a BaTiO_3_ thin film. The concurrent recording of electro-optic and piezo-optic mappings revealed that, far from the ideal uniformity, the ferroelectric and piezoelectric responses were strikingly inhomogeneous, exhibiting significant fluctuations over the scale of the micrometer. The optical methods here described are appropriate to study the variations of these properties simultaneously, which are of great relevance when ferroelectrics are downscaled to small sizes for applications in data storage and processing.

The electrical polarization in ferroelectrics can be reversed by the application of electric fields. This property is exploited in nonvolatile memories^1^ and ferroelectric tunnel devices^2^,^3^,^4^ to achieve bistable operation for digital processing and storage^5^,^6^. While the technological potential of ferroelectrics is beyond doubt, a deep understanding of the dynamics and the local response is crucial for the comprehension of the mechanisms underlying the retention and fatigue^7^. In such a context, piezoresponse force microscopy has been widely used to characterize ferroelectrics at small scales^8^,^9^,^10^,^11^,^12^,^13^,^14^,^15^,^16^. Alternatively, optics provides a way to image the ferroelectric response by measuring the birefringence arising from electro-optic effects^17^,^18^,^19^,^20^,^21^,^22^,^23^.

When it comes to applications requiring deep dimensional scaling, the device yield is critically dependent on the uniformity of the ferroelectric properties. In this regard, ferroelectrics that are constrained to nanometric lateral dimensions have been shown to exhibit size effects that promote spatially incoherent switching between the opposite polarizations states^9^. Obviously, this is a critical issue for the operation of ferroelectric devices since such effects limit the polarization dynamics of ferroelectric nanoregions, i.e., the polarization reversal may not be spatially coherent and be dependent on the frequency of the driving electric fields. Yet, beyond the particulars of the polarization dynamics, one may well also query about the spatial distribution of the ferroelectric polarization. For instance, it is known that defects may cause pinning of the polarization states that leads to an incomplete saturation in the hysteresis loops^9^,^24^. This begs the question of how the up or down polarization states are spatially distributed in the ferroelectric.

Our study, aimed at answering this question, exploited optics as a noninvasive way to peer into the local responses of both ferroelectric and piezoelectric properties. A bonus of using optics is that the ferroelectric properties can be probed without any restriction for the top electrodes –provided that they are thin enough to allow light transmitted through them –. This aspect is relevant, as it has been demonstrated that ferroelectric properties depend critically on the particular choice of the top-contact metal^25^,^26^. Not less important is the fact that polarized light is sensitive to both ferroelectricity and magnetism –the latter through magneto-optic effects–, thus being able to sense both degrees of freedom even at deeply buried interfaces, which is certainly a good asset when it comes to analyzing magnetoelectric coupling in composite systems^27^,^28^. Finally, the sensitivity of polarized light to strains expands the interest of optical imaging to the visualization of stress gradients in systems where flexoelectricity, even in centric materials, trigger polar responses^29^.

The experiments here described were carried out in an archetypical perovskite ferroelectric, namely, BaTiO_3_. In our approach, the analysis of the ferroelectric properties has been undertaken by exploiting the interaction of polarized light with matter subjected to electric fields (*E*). In these circumstances, an optical birefringence in a ferroelectric appears because of changes in the refractive index mediated by linear electro-optic (*r*_*EO*_) as well as piezo-optic (*r*_*PE*_) coefficients, i.e.^30^,^31^


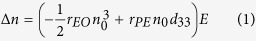


In this simplified equation, which disregards any tensorial properties, Δ*n* is generally a function of the wavelength of light, *n*_0_ is the refractive index at zero field and *d*_33_ is the longitudinal piezoelectric coefficient. The birefringence Δ*n* is associated to the changes of refractive index proportional to the internal electric field in the ferroelectric and gives way to a rotation of the polarization of light transmitted through the ferroelectric –typically in the range of the mrad for thin films–. There are two fundamentally different contributions to the birefringence Δ*n*. The first term in Equation 1 stems from linear electro-optics (EO) via the *r*_*EO*_ coefficients, also known as Pockels coefficients, caused by changes of the refractive index directly induced by electric fields. The EO term does only appear in noncentrosymmetric crystals, as is the case for any ferroelectric^30^,^31^ and, thus, to the first order, the EO-induced birefringence is proportional to the ferroelectric polarization. As the electro-optic signal can only occur for acentric systems, it is not affected by the kind of spurious artifacts that may mislead the interpretation of data^32^, offering optics an edge over other experimental approaches to characterize ferroelectricity. The second term in Equation 1 is caused by the piezo-optic or photoelastic effect (PE)^31^, which is the birefringence induced by strain field-induced deformations in the ferroelectric that, in turn, arise from the converse piezoelectric effect.

Equation 1 can be transformed in a way so as to reflect the dependence of Δ*n* on the ferroelectric polarization *P*, which is related to the applied electric field by *P* = χE, where χ is the linear electric susceptibility. On the other hand, the longitudinal piezoelectric coefficient is related to the ferroelectric polarization through *d*_33_ = 2*Q*χε_33_*P*, where Q is the electrostrictive coefficient and ε_33_ is the dielectric permittivity^33^,^34^. All in all, Equation 1 can be rephrased into


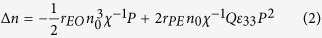


From Equation 2 we see that, whereas the electro-optic term is linear in the ferroelectric polarization, the piezo-optic contribution is quadratic. This observation implies a different parity relationship (odd versus even) with electric fields and, therefore, with the ferroelectric polarization. We have therefore exploited the distinctive parity of the EO (odd) and PE (even) terms with field to unravel the contributions related to electro-optics –linked to ferroelectricity – and to strain fields, associated to converse piezoelectricity.

With this in view, we have exploited electro-optic effects and strain driven birefringence to image the ferroelectricity and converse piezoelectric responses with diffraction-limited resolution. Using this method we analyzed the properties of a BaTiO_3_ thin film, and found that the electro-optic activity and the strain-related birefringence are strikingly far from the expected ideal uniformity, indicating that the ferroelectric and strain properties are inhomogeneous over the scale of the micrometer, with peak-to-peak fluctuations that, in the case analyzed here, reach up to about 20% around the averaged value. Our study also reveals that such inhomogeneity is rather sensitive to changes in temperature, becoming increasingly stronger as the temperature rises. We discuss at the end the possible origin of such inhomogeneous distribution of ferroelectric properties, as well as the implications that this non-uniformity may have for applications requiring ferroelectric devices.

## Results

### Sample preparation and characterization

**Figure 1:**
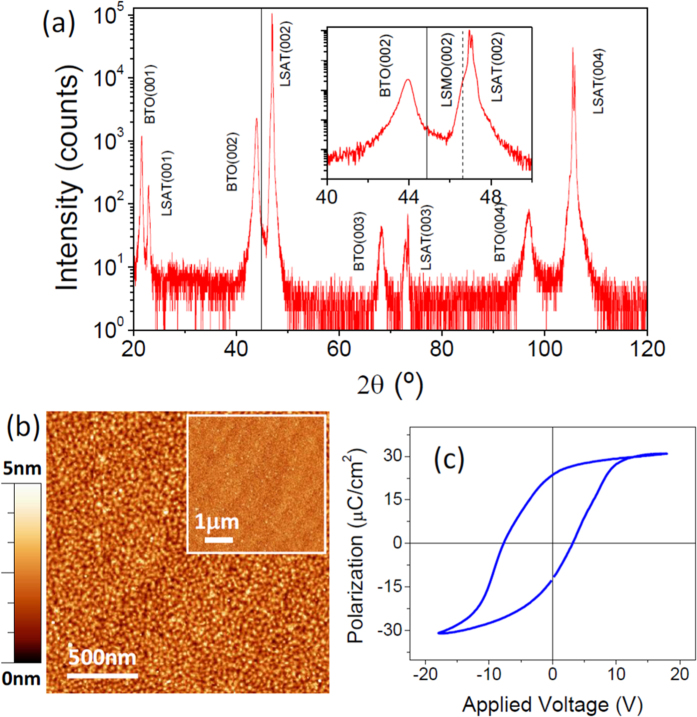
(**a**) XRD θ–2θ scan of the BaTiO_3_/La_2/3_Sr_1/3_MnO_3_/LSAT(001) sample. Peaks corresponding to reflections from BaTiO_3_ are indexed as BTO and those of La_2/3_Sr_1/3_MnO_3_ as LSMO. The inset in (**a**) shows a zoom around the BTO(002), LSMO(002) and LSAT(002) reflections. The vertical continuous line marks the position of the BTO(002) reflection for bulk BTO, whereas the dashed one indicates that of LSMO(002). (**b**) AFM topographic image (2 × 2 μm^2^) of the sample. The inset shows the AFM image over an area 5 × 5 μm^2^. (**c**) Ferroelectric polarization – voltage loop of the sample, measured at room temperature with LSMO as the bottom electrode and a Pt top electrode.

We studied a Pt (10 nm)/BaTiO_3_ (120 nm)/La_2/3_Sr_1/3_MnO_3_ (40 nm) trilayer grown on a (001)-oriented (LaAlO_3_)_0.3_-(Sr_2_AlTaO_6_)_0.7_ (LSAT) substrate. Details on sample preparation are given in the Methods section. X-ray diffraction (XRD) θ–2θ scans (Fig. 1a) showed (*00* *l*) reflections from the LSAT substrate, BaTiO_3_ and La_2/3_Sr_1/3_MnO_3_ layers, with absence of any spurious phases or other orientations. The lattice parameters of La_2/3_Sr_1/3_MnO_3_ and the LSAT substrate are very close: 3.868 Å the former and 3.875 Å the latter. Thus, in Fig. 1a the diffraction peaks of the La_2/3_Sr_1/3_MnO_3_ film are overshadowed by their close proximity to the high intensity LSAT peaks. From the position of the BaTiO_3_(002) peak (inset of Fig. 1a) we determined the out-of-plane lattice parameter *c* = 4.12 Å, which is expanded with respect to the BaTiO_3_ bulk value (*c* = 4.038 Å)^35^,^36^. Within the sensitivity of the measurement, *a*-oriented domains were not detected. The surface morphology of the BaTiO_3_/La_2/3_Sr_1/3_MnO_3_ bilayer was analyzed by atomic force microscopy (AFM) topographic images and is presented in Fig. 1b. The 2 × 2 μm^2^ image shows morphology of islands having a small lateral size of a few tens of nanometres. In spite of the presence of islands, the surface is quite flat, with root-mean-square (rms) roughness of 7 Å, and the morphology of terraces and steps can be barely appreciated in the 5 × 5 μm^2^ image presented in the inset. Nearly circular platinum (Pt) contacts with area ~0.2 mm^2^ were deposited on top of the BaTiO_3_ thin film for ferroelectric characterization. The thickness of the Pt electrodes was thin enough (*t*_*Pt*_ ≈ 10 nm) so that they were partially transparent to light, allowing the measurement of the electro-optic and piezo-optic responses of the ferroelectric BaTiO_3_ layer underneath. Panel (c) shows the polarization – voltage curve (Fig. 1c), which exhibits a ferroelectric hysteresis loop (see Methods for details), with spontaneous polarization P ≈ 24 μC/cm^2^, comfortingly close to the values reported for bulk^37^.

### Optical characterization

**Figure 2:**
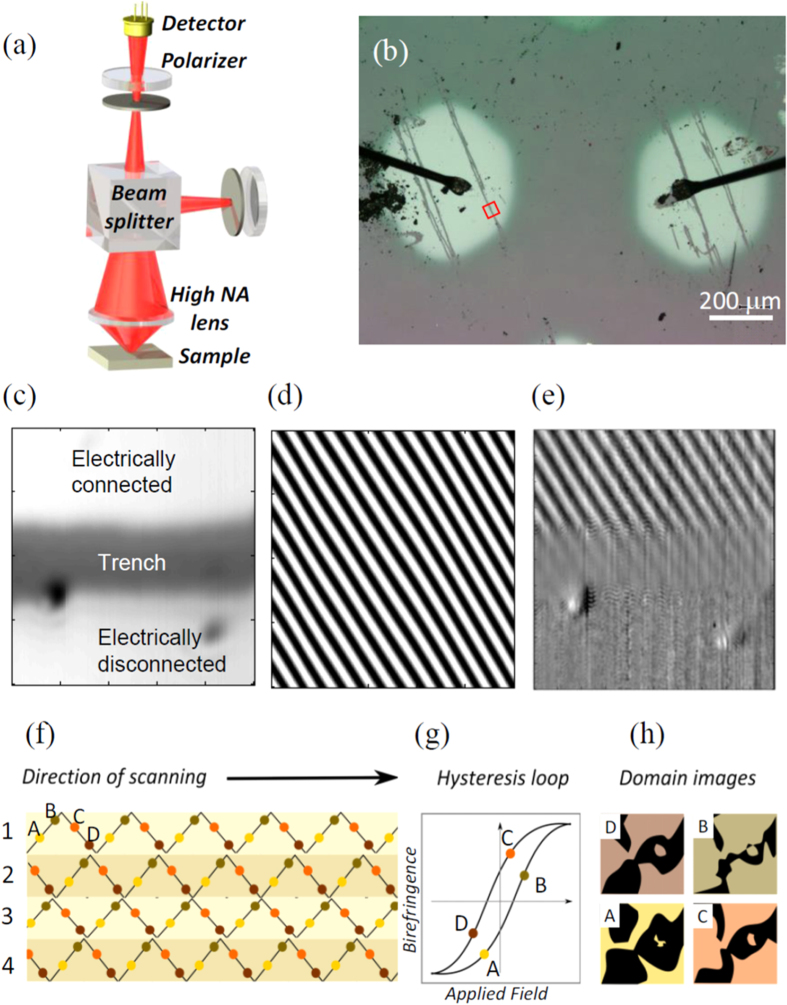
(**a**) Schematics of the confocal optical arrangement used to map the birefringence. (**b**) Micrograph showing two Pt electrodes. The small red square encloses an area stretching across a trench separating a small area connected to a wire from another that was electrically isolated. (**c**) Zoom of the region at the center of the red square in (**b**). Panel (**d**) shows the spatial distribution of electric fields –generated by a triangular-wave source– applied on this area. Dark/light stripes correspond to positive/negative values of the field, respectively. (**e**) Spatial distribution of the birefringence in response to the electric field pattern shown in (**d**). The optical contrast emerges as response to the applied electric field pattern. Panels (**f–h**) exemplify schematically the procedure for the optical imaging. For illustration, particular values of the electric field are indicated symbolically in the triangular wave of panel (**f**), color-coded and labelled with letters. Figurative representations of a birefringence hysteresis loop as well as the spatial distribution of birefringence are shown in panels (**g**,**h**), respectively. Labels and color codes of panels (**g**,**h**) are in correspondence with the labeled dots in panel (**f**).

To achieve diffraction-limited lateral resolution we adapted a confocal optical arrangement to measure the rotation of the polarization of light resulting from the birefringence in the ferroelectric BaTiO_3_ thin film (see the sketch of Fig. 2a and Methods for a detailed description of the optical characterization). We first analyzed the birefringence measured all over a small region with size of about 5 × 5 μm^2^ on the Pt contact, located at the center of the small red square in the optical picture displayed in Fig. 2b. In the middle of this area we intentionally scratched the surface of the Pt top contact to create a deep trench, separating a zone that was contacted with a wire from another that was electrically isolated. These two subregions are clearly seen in the optical mapping shown in Fig. 2c. The purpose of defining these two subareas was to ensure that any contrast in the birefringence measured in our microscope was caused by the ferroelectric in response to an external electric field. In other words, the varying electric fields would produce a contrast in the image scanned over the electrically connected area, whereas no contrast should be observed in the electrically isolated area.

To check this issue, the beam spot was moved by successive line scans over the region under scrutiny. Over each of these lines, the beam was positioned at equally spaced locations, and at each position the rotation of the polarization of light was measured while an electric field *E* was applied between the Pt top contact and the La_2/3_Sr_1/3_MnO_3_ bottom electrodes. A triangular wave source was used to generate the electric field *E* while simultaneously the beam light was swept over the sample. For the positive slopes on the triangular wave, the field was increased for successive beam locations over a line scan, until a maximum was reached at the top of the triangular wave. Subsequently, the negative slopes on the triangular profile were accessed, and the electric field was decreased and eventually reversed its sign for consecutive locations over the line scan. With this triangular wave we generated a streaked pattern representing the values of the applied fields at each location over the analyzed area. This is shown in Fig. 2d, where the intensity of the dark and light stripes corresponds to locations on the scanned surface where the fields were positive and negative, respectively.

The application of the triangular-wave electric field pattern produced, as expected, a contrast in the birefringence in the electrically connected area of the sample, but not in the isolated area. That is to say, when we mapped the birefringence measured over the analyzed region, a streaky pattern with crests and troughs –corresponding to maxima and minima of birefringence– was observed in the subarea that was electrically connected. Figure 2e shows that this banded pattern exhibited the same spatial modulation as that of the applied fields –compare Fig. 2d,c–. On the contrary, even with the same applied triangular-wave field pattern, the optical contrast was smeared out in the region of the trench and was completely absent over the electrically isolated area –compare Fig. 2e,c–. We conclude, thus, that the birefringence was modulated only in the area in which electric fields could be effectively applied and, hence, we exploited the susceptibility of the optical signal to electric fields to probe the ferroelectric properties of the BaTiO_3_ film.

To do so, as aforementioned, the region under scrutiny was analyzed by carrying out successive linear beam scans, labelled 1–4 in the schematics of Fig. 2f, while the birefringence was recorded for locations separated typically about 5 nm. The local electro-optic and piezo-optic responses were obtained after averaging over small areas with size compatible with the diffraction-limited resolution (for more details see *Image processing* in Methods). From the collection of data, two different kinds of information could be extracted. To illustrate the procedure, we indicate selected points on the triangular-wave pattern of Fig. 2f, which for the sake of clarity are coded in different colors and are labelled with letters. A first source of information regards the extraction of hysteresis loops from the birefringence measured over a given area. This is shown in the birefringence loop sketched in Fig. 2g; letter-labeled dots on this loop refer to the values of the electric field selected in the triangular-wave pattern of Fig. 2f. A second source of information is provided by mapping the values of the birefringence over the analyzed region for different values of the electric field, as shown in the sketch of Fig. 2h. Put differently, we imaged –with diffraction-limited lateral resolution– the spatial distribution of the birefringence measured at constant applied electric field. Figure 2h is a figurative representation of a possible evolution of birefringence maps as a function of the field values selected in Fig. 2f.

### Mapping ferroelectricity and strain from birefringence

**Figure 3:**
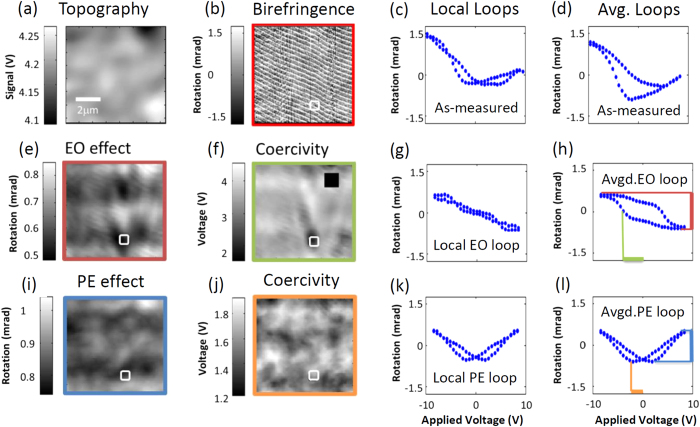
(**a**) Topography chart of the Pt top electrode (**b**) Spatial distribution of the birefringence modulated by the electric field triangular-wave pattern. (**c**) As-measured hysteresis loop extracted from birefringence recordings measured inside the white square box of panel (**b**). (**d**) Hysteresis loop obtained by averaging over all data in this area. (**e**) Mapping of the electro-optic signal at the highest field, obtained from the magnitude of the loops (red line in Fig. 3h). In (**f**) the distribution of coercive fields (green line in Fig. 3h) is shown, whereas (**g**,**h**) show the local and averaged electro-optic loops. (**i**) Magnitude of the piezo-optic response (blue line in Fig. 3l) imaged at the highest field. The distribution of coercive fields, defined by the deflection peaks in the piezo-optic loops (orange line in Fig. 3l) is shown in (**j**) –see (**k**) for the piezo-optic local loop measured in the square box of (**b**) and see (**l**) for the averaged loop–.

Yet, in the actual experiments, the birefringence was affected by small fluctuations of the electrode thickness. Thus, in order to have access to the intrinsic ferroelectric properties of the BaTiO_3_ thin film, the extrinsic influence of the topographic features of the Pt was detected and suppressed. We took advantage of the fact that the birefringence associated to topography induces a vertical offset in the loops (see Figure S1 of the Supplementary Information). Bearing this in mind, we centered the hysteresis loops after averaging the signals at remanence over the upper and lower branches. Therefore, by mapping the vertical offset of the loops we could obtain a topographic map of the Pt surface, which is shown in Fig. 3a. After subtracting this contribution, the intrinsic birefringence in response to the triangular wave field pattern could be imaged all over the analyzed area, as shown in Fig. 3b. Following the protocol outlined above, the spatial distribution of the measured birefringence was used either to extract local hysteresis loops at every spot –see one example in Fig. 3c– or to obtain a loop averaged all over the region under analysis, as shown in Fig. 3d.

The local and averaged loops shown, respectively, in Fig. 3c,d, are significantly different from those obtained by measuring the displacement currents in the standard polarization – electric field characterization (Fig. 1c). The reason is that both electro-optic (EO) effects –linked to ferroelectricity– as well as piezo-optic terms from the converse piezoelectricity (PE) add up to the recorded birefringence. The EO and the PE terms were deconvoluted from the as-measured birefringence by mathematical (anti)-symmetrization. This follows from the fact that the EO and PE components have odd and even parity with respect to the applied fields, i.e., *θ*^*EO*^(*E*) = −*θ*^*EO*^(−*E*) and *θ*^*P*E^(*E*) = *θ*^*P*E^(−*E*), respectively. Note that although information on the imprint is lost in this process, other parameters –saturated electro-optic signal, absolute coercive field difference (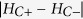) and strain-driven birefringence– stay unaltered.

The (anti)-symmetrization processing of the recorded birefringence data allowed independent access to the electro-optic and piezo-optic contributions. In particular, the protocol delineated above allowed us to map the electro-optic signal at the highest applied field, close to saturation, for which the spatial distribution is displayed in Fig. 3e. At the same time, both local (Fig. 3g) and averaged (Fig. 3h) electro-optic loops were obtained. From the local loops, the spatial distribution of coercive fields could be also imaged, as shown in Fig. 3f. In this image, the black square dot refers to a region where the loops are extremely flat in shape; a similar situation –although to a lesser degree- occurs also for the loop shown in Fig. 3g. Therefore, in the region signaled by the square block in Fig. 3f the loop is crossing the vertical line at a very long interval, so that no meaningful coercivity values are obtained were obtained for that particular region. Conversely, Fig. 3i displays the imaging of the piezo-optic birefringence measured at the highest applied field, whereas Fig. 3k,l show the local and averaged piezo-optic hysteresis loops, respectively. The information was completed by imaging in Fig. 3j the coercive fields defined by the deflection peaks in the piezo-optic butterfly-shaped loops recorded at each location of the analyzed area.

A visual inspection of Fig. 3e,i revealed that the distribution of electro-optic and piezo-optic responses was far from the ideal uniformity, and they rather exhibited significant variations all over the region under consideration. Indeed, these mappings disclosed peak-to-valley modulations of about 20% around the average values. For instance, the electro-optic birefringence induced rotations of light polarization that changed over the analyzed region in a range between approximately 0.5 mrad and 0.83 mrad (Fig. 3e), while the values of the piezo-optic signal were comprised within an interval extending from around 0.76 mrad to 1.03 mrad (Fig. 3i). An extreme example of the effect of these fluctuations is perceptible upon examination of the electro-optic hysteresis loop measured at the position signaled by the rectangular white boxes in Fig. 3. For this specific region, the local and averaged electro-optic loops were strikingly dissimilar –compare Fig. 3g,h–, further bearing out the conspicuous non-uniform distribution of ferroelectric properties.

**Figure 4:**
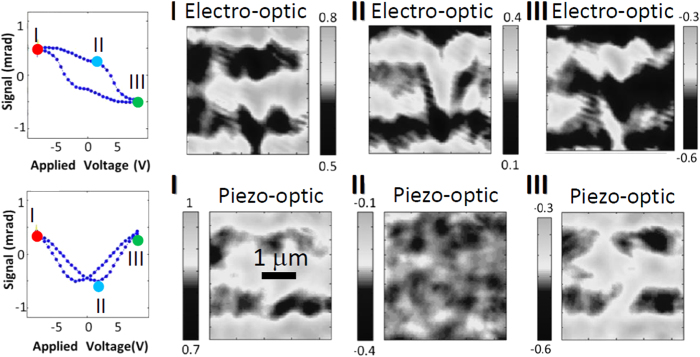
The spatial distribution of the electro-optic and the piezo-optic responses are displayed at different values of the electric field. The figures show the mappings corresponding to the largest positive and negative applied fields, designated as points I and III on the hysteresis loops, respectively. Mappings are also shown for intermediate fields, close to the coercive field designated as II on the hysteresis loops. Vertical grayscale bars next to each mapping indicate the rotation (in mrad) with values above (light) and below (dark) the average. The resulting images emphasize the inhomogeneous distribution of the electro-optic and piezo-optic responses.

The inhomogeneous spatial distributions of the electro-optic and piezo-optic birefringence were also charted for different values of the electric field. This is shown in Fig. 4: light regions correspond to the zones where the measured birefringence was above the average value, whereas dark areas are related to values below the average. Again, the overall picture displayed in Fig. 4 is that the nearly saturated electro-optic and piezo-optic terms to the birefringence exhibit significant modulations –by around 20% – over the scale of the micrometer. Figure S2 of the Supplementary Information provides a graphical representation of the typical distribution of the electro-optic recordings over the scanned areas.

**Figure 5:**
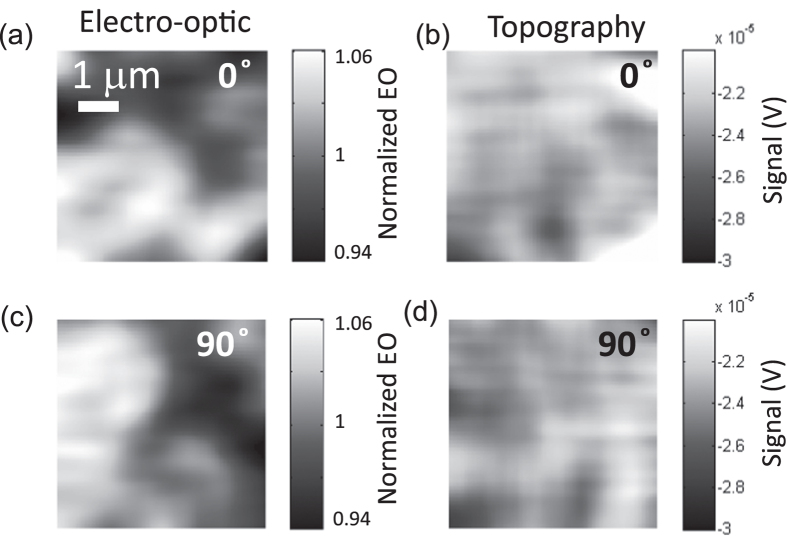
Birefringence imaged over the same surface after scanning along two orthogonal directions, along 0° and 90°, respectively. Electro-optic mappings are shown in (**a**,**c**), whereas the topography is shown in (**b**,**d**). A visual inspection demonstrates that mappings are consistent with a rotation by 90°, and confirm the intrinsic character of the fluctuations in the electro-optic responses of the BaTiO_3_ thin film. The units of the electro-optic mapping are normalized to the value obtained by averaging over all the scanned area.

The intrinsic character of the observed inhomogeneity was confirmed by scanning the surface along two orthogonal directions, denoted as 0° and 90°, respectively, see Figure 5. In this experiment the same area was raster-scanned twice, with the only difference that the beam spot was scanned along two orthogonal directions. In a first experiment, the beam spot was scanned along a direction that we define as a reference (hence we refer to such an image as scanned along 0°). In a subsequent experiment, we changed the settings of the piezoactuators that moved the sample to scan lines along an orthogonal direction oriented at 90° with respect to the former scanning lines. Thus, the experiment was formally equivalent to rotate the sample by 90° between successive mappings. In consequence, if the spatial modulations of the electro-optic response are intrinsic to the sample, then specific features observed in the mappings should also be rotated by 90° when comparing the two mappings. The rotation experiment was carried out over an area located on a different place than the one discussed in previous paragraphs, so that the fluctuation pattern observed in Fig. 5 differs substantially from those shown in Figs 3 and 4. A visual inspection of the electro-optic birefringence (Fig. 5a,c) and topography (Fig. 5b,d) clearly reveals that the characteristic variations are rotated 90° to each other. This consistency under rotation unambiguously demonstrates that the fluctuations of the electro-optic and piezo-optic birefringence are intrinsic to the sample.

**Figure 6:**
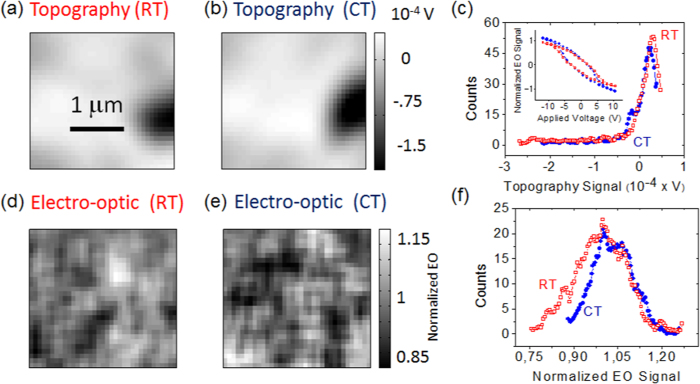
The topography measured from optical images recorded at ≈9 °C (CT) and at room temperature ≈25 °C (RT) are shown in panels (a) and (b), respectively. Both images were taken at the same location, hence the similitude of both mappings. The distribution of topography data measured at CT and RT is displayed in panel (**c**). The inset of panel (**c**) shows the electro-optic hysteresis loops averaged over the whole scanned at CT and RT. The electro-optic mappings recorded at CT and RT are displayed in panels (**d**,**e**), respectively. Histograms corresponding to the electro-optic mappings are plotted in panel (**f**), which depict the frequency (number of counts) for each value of the electro-optic signal measured at the highest applied voltage. The electro-optic values are normalized to the average recorded value.

Finally, we assessed the effect of temperature on the spatial distribution of the electro-optic signals (see Methods for technical details). For that purpose, the birefringence was mapped over the same area at two different temperatures, namely at T ≈ 9 °C (cold temperature, CT) and T ≈ 25 °C (room temperature, RT). The topography extracted from these images remained, as expected, essentially unchanged, as shown in Fig. 6a,b; unsurprisingly, the histograms plotting the frequency distribution of the topography data (Fig. 6c) overlapped. While the resemblance of the topography ensured that the same region was scanned for both experiments, the electro-optic mappings recorded at CT and RT disclosed remarkable differences. First, the hysteresis loops averaged over the scanned areas (inset of Fig. 6c) showed that the electro-optic signal decreased when rising the temperature, an effect that we attribute to pyroelectricity as the ferroelectric was heated from cold to room temperature. Secondly, the electro-optic responses at CT (Fig. 6d) and RT (Fig. 6e) exhibited significant changes in their spatial distribution as a function of the temperature. Indeed, the statistical analysis of the optical mappings showed that the frequency distribution of the electro-optic responses was perceptibly narrower for measurements done at the cold temperature with respect to those at room temperature (Fig. 6f), indicating that even rather moderate temperature variations (about 15 °C) leave a noticeable imprint on the magnitude of the spatial inhomogeneity of ferroelectric properties. In the Supplementary Information (Figure S3) we discuss further experiments that show the amplification of the electro-optic spatial inhomogeneity in response to higher temperatures.

## Discussion

To sum up, by monitoring the spatial distribution of the birefringence we had access to the individual contributions of the ferroelectric and piezoelectric responses and their evolution with the applied electric fields. The diffraction-limited resolution of the optical imaging enabled us to uncover remarkable variations of the properties of the BaTiO_3_ thin film over the scale of a micrometer. Several scenarios can be argued to explain the observed inhomogeneity. To begin with, although X-ray diffraction data indicate that BaTiO_3_ is *c*-oriented, a fraction of *a*-oriented domains, undetected within the sensitivity of the X-ray experiments, cannot be excluded. Should that be the case, the contributions from *a*- and *c*- domains might not affect in the same way the electro-optic and piezo-optic signals. Alternatively, even if *c*-orientation is preserved throughout, inhomogeneous strain may lead to local changes in ferroelectricity that could also account for the observed variations in the optical birefringence. Indeed this latter scenario may conform better to the observed increase of the spatial inhomogeneity of the electro-optic response as the temperature rises. Nonuniform strain distribution may be promoted by the fact that the lattice parameters of the LSAT substrate and BaTiO_3_ film are remarkably different, i.e., *a*_*LSAT*_ ≈ 3.868 Å and *a*_*BTO*_ ≈ 3.994 Å (and *c*_*BTO*_ ≈ 4.038 Å), respectively. In spite of the large mismatch of about 3.2%, the out-of-plane parameter of BaTiO_3_ is strongly expanded (*c*_*BTO*_ ≈ 4.12 Å). Such large strain could be favored by local off-stoichiometry (e.g., by oxygen vacancies) or point defects promoting a spatially inhomogeneous ferroelectricity. Indeed, in a similar context, the generation of point or extended defects is believed to promote a spatially inhomogeneous distribution of strain and local ferroelectricity in SrTiO_3_ crystals^38^.

We then suggest that the space distribution of strain fields may have a deep impact for applications of ferroelectric materials. A particularly illustrative example is the application of ferroelectrics for data storage. Nonvolatile memories based on ferroelectric tunnel junctions represent a promising route to store information as ferroelectric polarization^39^. Indeed, junction devices with sizes down to tens of nanometers have been demonstrated^3^,^40^,^41^,^42^, in which the readout is done by measuring the electrical resistance that, in turn, is determined by the electric polarization. In the light of our experiments, highly strained states, suggested to enhance the properties of the ferroelectric barriers in the tunnel junctions^3^,^39^, may be influential in the space distribution of the digital binary states. As the readout is expected to be very sensitive to the electric polarization, any space modulation of the ferroelectric properties would thereof have strong influence in the yield performance, given that arrays of nominally identical storage cells extend over distances beyond the micron scale.

## Methods

### Optical Setup

To measure the birefringence, the changes in the linear polarization state of light were detected using an attoCFM I confocal microscope from Attocube Systems AG. For that purpose TEM_00_ mode of light coming from a red laser He-Ne laser (λ = 632.8 nm) was let through a polarizer and, after deflection by a beamsplitter, it was focused on the sample surface by means of a high-numerical aperture objective lens (see the sketch in Fig. 2a). After reflection on the sample, the light was passed through a pinhole filtering out all the out-of-focus light and was collected by a detector after going through a second polarizer (analyzer) with the optical axis set at 90° with respect to the first polarizer (Fig. 2a). Prior to measurements, the orthogonal polarization sets of the polarizer and analyzer were ensured by fine-tuning the intensity collected at the detector to the lowest value. In the experiments, any rotation of the polarization plane is detected as an unbalance of the initial conditions that, following the Malus’ law, generate changes of the intensity of light that goes through the analyzer.

### Raster-scanning

The sample was mounted on a holder attached to piezoactuators that translated the specimen across the *x-y* (horizontal) plane, as well as along the vertical direction (*z*). With the help of the piezoactuators, the beam spot was raster-scanned over the surface defining a mesh of *N*_*V*_ × *N*_*H*_ points determined from the number of vertical (*N*_*V*_) and horizontal (*N*_*H*_) pixels. By that we mean that the beam was swept horizontally left-to-right at a steady rate and, then, it moved back to the left at which point it swept out to the next line. In this raster-scanning operation, electric fields were applied, modulated according to a triangular wave profile with a periodicity 1/*v*_e_ ≈ 10 ms. A typical image, with area 5 × 5 μm^2^, consisted of *N*_*V*_ × *N*_*H*_ ≈ 10^3^ × 10^3^ pixels. While scanning, the spot stopped at each location for a certain amount of time –the acquisition time– in which the signal that reached the detector was integrated before moving to the new location on the surface. The frequency of this scanning was limited by the refresh rate of the detector (which in our case was 1 MHz for the Newport large area balanced photoreceiver, model: 2307). Increasing the acquisition time is beneficial to increase the signal-to-noise ratio. Yet a compromise is needed, since in a typical image is composed of millions of points, so that the time per loop has to be kept as short as possible. Additionally, increasing too much the acquisition time comes with troubles related to temperature and piezo-driven drifts. After extensive analysis of all these aspects, we found that an acquisition time of 100 microseconds was a good compromise.

### Image processing

In order to obtain the local ferroelectric and strain responses, the birefringence loops were averaged over small areas with size compatible with the diffraction-limited resolution *Δr* ≈ *0.44λ/(2* × *NA)*^43^, which, for the wavelength used in experiments (*λ* = 632.8 nm), is *Δr* ≈ 409 nm. An evaluation of the diffraction-limited lateral resolution is presented in Figure S4 of the Supplementary Information. For the image processing, the entire set of pixels (about 10^6^) was subdivided into smaller cells, with a typical cell size of around 100 × 100 pixels each, so that the surface mapped by a cell was comparable to the diffraction-limited resolution. Each of these cells included a large amount of pixels (~10^4^), each of them associated with a given value of applied field and birefringence. Therefore, it was possible to identify a number of birefringence recordings for a particular value of the electric field. Hysteresis loops were extracted by averaging over the birefringence recordings for each value of the electric field over the processing cell.

### Optical Imaging at Different Temperatures

A Peltier thermoelectric module was used for measurements done as a function of temperature. By changing the temperature, two effects may affect the optical characterization. A first effect is related to the thermal expansion of the piezoactuators or the sample holder itself. Prior to any measurement, the position *z* is adjusted so that the specular reflection of the beam spot on the sample surface –as seen through a CCD camera attached to a microscope lens –is as symmetric as it can be. This ensures that the beam spot is well aligned along all the optical axes, including polarizers, analyzers, beamsplitters and the high-numerical aperture lens. However, upon changing the temperature, the beam spot was out-of-focus at the new temperature, so that the shape of the beam spot was distorted from the initial symmetric shape. To correct these effects, after letting enough time to settle the temperature to a stable value, the vertical position of the piezo actuators was finely adjusted to put the beam spot back again in focus.

A second effect was related to thermal drifts that caused that the surfaces actually mapped were not the same for images taken at two different temperatures. Drifts typically of 1–2 μm were usually found for measurements done at different temperatures. To correct this effect and make sure that we compared mappings over the same area, we used auto-correlation algorithms on the topography images that were acquired simultaneously with the electro-optic signal. Auto-correlation algorithms were, for instance, used to ensure that the electro-optic response shown in Fig. 6c,d were obtained over the same area, as displayed in Fig. 6a,b.

### Sample Growth

The BaTiO_3_ (120 nm)/La_2/3_Sr_1/3_MnO_3_ (40 nm) bilayer was grown by pulsed laser deposition on a LSAT(001) substrate. The deposition temperature, laser frequency and oxygen pressure during growth were 700 °C, 5 Hz and 0.02 mbar for BaTiO_3_, and 725 °C, 2 Hz and 0.2 mbar for La_2/3_Sr_1/3_MnO_3_. Pt contacts –with area ≈0.2 mm^2^– were deposited on top of the BaTiO_3_/La_2/3_Sr_1/3_MnO_3_ heterostructure by sputtering through a mask. The Pt thickness ≈10 nm was suitably small to ensure that enough light was transmitted through it.

### Ferroelectric characterization

The ferroelectric polarization of the BaTiO_3_ layer was measured using a TF Analyser 2000 from AixAcct Co. The ferroelectric hysteresis loops were extracted using the dynamic leakage current compensation (DLCC) method and the frequency of the measurements ranged from 100 Hz up to 500 Hz.

## Additional Information

**How to cite this article**: Vlasin, O. *et al.* Optical Imaging of Nonuniform Ferroelectricity and Strain at the Diffraction Limit. *Sci. Rep.* **5**, 15800; doi: 10.1038/srep15800 (2015).

## Supplementary Material

Supplementary Information
