# Supplementary material for: Optical Imaging of Nonuniform Ferroelectricity and Strain at the Diffraction Limit
Source: Sci Rep. 2015 Nov 2;5:15800. doi: 10.1038/srep15800 (PMC4629134; doi:10.1038/srep15800)
Supplement: Supplementary Information [file srep15800-s1.doc]

**Optical Imaging of Nonuniform Ferroelectricity and Strain at the Diffraction Limit**

Ondrej Vlasin, Blai Casals, Nico Dix, Diego Gutiérrez, Florencio Sánchez, Gervasi Herranz^*^

Institut de Ciència de Materials de Barcelona (ICMAB-CSIC), Campus de la UAB,

08193 Bellaterra, Catalonia, Spain

* gherranz@icmab.cat

SUPPLEMENTARY INFORMATION.

Information is provided on the effect of topography on the measured optical signal and the statistical analysis of electro-optic mappings and their dependence on temperature.

**Figure S1**. (a) The confocal principle relies on letting in-focus light to reach the sample and then enabling only in-focus reflected light to reach the detector. This is done by positioning the sample in the focal plane of a high numerical aperture objective, whereupon light reflected from the surface is passed through a pinhole filtering out all out-of-focus light. To analyse the rotation of the polarization of light, a polarizer is placed just before the detector. The effects of topography are schematically depicted in panel (b). For the sake of simplicity, the confocal arrangement is not displayed, and only the detector and polarizer are shown. The intensity of light from the incident beam (IB) is reflected off the surface and then goes through a polarizer (P) before reaching the detector (D). As the beam spot is scanned over the area under analysis, it may well happen that occasionally it stumbles upon a topographic feature. For instance, when the surface is not perfectly flat, the light reaching the detector is slightly defocused, inducing changes of intensity that can be mistaken as an intrinsic rotation of light. As a consequence, the topography induces a vertical shift in the hysteresis loops as the applied electric field is cycled around, as shown in panel (c). Since this vertical offset depends on the location on the surface, it can be used to extract topographic features with diffraction-limited resolution.


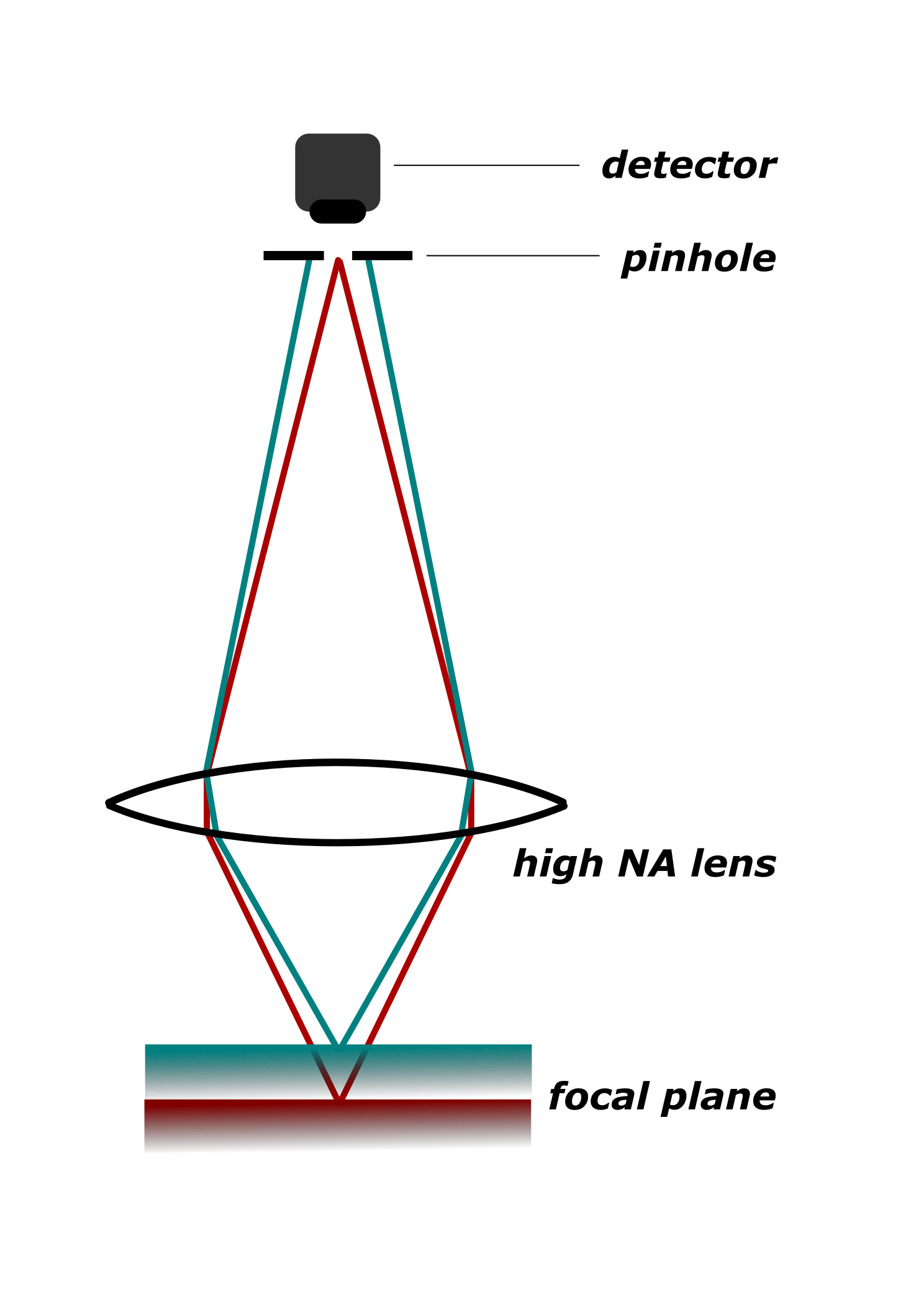


(a)

(b)

(c)

**Figure S2**. (a) Electro-optic mapping of the BaTiO_3_ thin film. (b) Histogram showing the distribution of data, indicating the frequency (counts) for each interval of electro-optic values. The step width is taken equal to ~ 6.9 × 10^-7^ V. In this particular case, the electro-optic recordings span within an interval of ~ 0.29 × 10^-4^ V, i.e., over a range of around 17.8 % of the arithmetic mean (which is ~ 1.628 × 10^-4^ mV).

(a)

(b)

**Figure S3**. The spatial distribution of the electro-optic response and hysteresis loops was investigated by mapping the optical response at three different temperatures, namely cold (CT ≈ 9ºC), room (RT ≈ 25ºC) and hot (HT ≈ 60ºC) temperatures. In a first experiment, we carried two consecutive scan runs, each of one consisting in measuring first at ambient temperature and subsequently after cooling down to CT. The electro-optic histograms and hysteresis loops corresponding to these measurements are shown in panels (a) and (b), respectively. Additionally, in a separate experiment, we also measured the electro-optic data distribution and hysteresis loops measured first at RT and then at HT, see panels (c) and (d). The inspection of these data reveals that the magnitude of the electro-optic hysteresis loop decreases as the temperature rises, see panels (b) and (d). On the other hand, as the temperature increases, the inhomogeneous spatial distribution of the electro-optic response is heightened, as revealed by the broader data distribution shown in the histograms of panels (a) and (c).

**Figure S4**. To test the spatial resolution of our confocal microscope, we took images of a grating consisting of gold grooves with a periodicity of slightly above 1 micron. This sample was done by using e-beam lithography in conjunction with a lift-off stage to define the grooves. (a) Shows the image of the grating as observed by scanning electron microscopy. The image in the main panel of (b) displays the image as obtained by our confocal microscope. This Figure shows a striped structure of dark and light bands, in correspondence with the topography shown in (a). In order to better compare the image by SEM and that obtained by the confocal microscope we convoluted the image by SEM using a Gaussian filter with a width of 500 nm, close to the expected lateral resolution limited by diffraction. As seen in the inset of (b), the correspondence of the Gaussian-filtered SEM image and the mapping as measured by the confocal optics is excellent. This proofs that the images taken by the confocal system are compatible with optical imaging working at the diffraction limit.

(a)

(b)
